# Novel Tools to Analyze the Function of *Salmonella* Effectors Show That SvpB Ectopic Expression Induces Cell Cycle Arrest in Tumor Cells

**DOI:** 10.1371/journal.pone.0078458

**Published:** 2013-10-21

**Authors:** Beatriz Mesa-Pereira, Carlos Medina, Eva María Camacho, Amando Flores, Eduardo Santero

**Affiliations:** Centro Andaluz de Biología del Desarrollo/ CSIC/ Universidad Pablo de Olavide/ Junta de Andalucía. Departamento de Biología Molecular e Ingeniería Bioquímica, Seville, Spain; Innsbruck Medical University, Austria

## Abstract

In order to further characterize its role in pathogenesis and to establish whether its overproduction can lead to eukaryotic tumor cell death, *Salmonella* strains able to express its virulence factor SpvB (an ADP-ribosyl transferase enzyme) in a salicylate-inducible way have been constructed and analyzed in different eukaryotic tumor cell lines. To do so, the bacterial strains bearing the expression system have been constructed in a ∆*purD* background, which allows control of bacterial proliferation inside the eukaryotic cell. In the absence of bacterial proliferation, salicylate-induced SpvB production resulted in activation of caspases 3 and 7 and apoptotic cell death. The results clearly indicated that controlled SpvB production leads to F-actin depolimerization and either G1/S or G2/M phase arrest in all cell lines tested, thus shedding light on the function of SpvB in *Salmonella* pathogenesis. In the first place, the combined control of protein production by salicylate regulated vectors and bacterial growth by adenine concentration offers the possibility to study the role of *Salmonella* effectors during eukaryotic cells infection. In the second place, the salicylate-controlled expression of SpvB by the bacterium provides a way to evaluate the potential of other homologous or heterologous proteins as antitumor agents, and, eventually to construct novel potential tools for cancer therapy, given that *Salmonella* preferentially proliferates in tumors.

## Introduction

*Salmonella enterica* serovar Typhimurium (*S. Typhimurium*) is probably the pathogen that has been most extensively studied and exploited as an anti-tumor agent. It can invade different host cells, such as epithelial cells, macrophages and dendritic cells. Most importantly, *Salmonella* is capable of preferentially colonizing and proliferating in solid tumors to levels nearly 1000-fold higher than normal tissue, a situation that usually results in tumor growth inhibition [1]. Additionally, *Salmonella* is not only able to colonize large solid tumors, but also to accumulate in metastases when systemically administered [2,3]. The genetic manipulation of *Salmonella* is well developed and a variety of attenuated strains with mutations that render the bacteria safe for the host have been characterized [4,5]. The administration of attenuated *Salmonella* strains expressing different anti-tumor agents has been used in recent years with promising results in tumor regression [6–9].

After ingestion into the digestive tract, *Salmonella* induces macropinocitosis by epithelial cells through the injection of bacterial effector molecules that manipulate the host cytoskeleton [10]. This injection is mediated by the Type Three Secretion System (TTSS) encoded in the *Salmonella* pathogenicity island-1 locus (SPI-1). Inside the eukaryotic cell, bacteria remain enclosed in a membrane-bound vacuole termed Salmonella-containing vacuole (SCV). Effectors translocated by this TTSS and by a second TTSS (TTSS-2), encoded by the SPI-2 locus, contribute to the intracellular survival and replication of the bacteria (reviewed in 11). Once established inside epithelial cells, *Salmonella* is able to replicate and induce apoptosis after 18-24h [12,13].

Many *Salmonella* serovars, such as *S. Typhimurium*, carry an additional locus called *spv* [14] encoded by the *Salmonella* virulence plasmid (or chromosomally in some strains) that enhances virulence in animals and humans [14–18]. This locus encodes, among others, the SpvB protein, whose C-terminal domain confers ADP-ribosyl transferase activity [19,20]. This activity covalently modifies G-actin monomers thus preventing their polymerization into F-actin filaments, which causes the loss of the eukaryotic actin cytoskeleton [18,21–23]. These results have been shown using different approaches, such as adding purified SpvB protein to cell lysates, transfecting epithelial cells and macrophages to transiently express the protein, or infecting macrophages and epithelial cells with different *Salmonella* SpvB mutants to analyze their efficiency in depolymerizing actin. It is thought that SpvB is delivered into the eukaryotic cytosol via the SPI-2 TTSS [18,23–25] and that both the SPI-2 TTSS and SpvB are required for the late apoptosis produced by *Salmonella* in macrophages and epithelial cells [13,16]. However, the mechanism connecting SpvB to apoptosis induction remains unknown. 

In recent years, the use of compounds that inhibit or prevent actin polymerization to reduce the growth of several tumor cell lines has been investigated [26,27]. Cytotoxic agents that interfere with cytoskeleton dynamics have a recognized potential utility in the cancer treatment. For instance, natural toxins such as pectenotoxin 2, isolated from dinoflagellates, have been shown to have a potent apoptosis inducing effect on human cancer cells lines [28], together with G2/M arrest and endoreduplication [28–31]. Since purified SpvB is unable to enter eukaryotic cells [22], here we have used *Salmonella* to express SpvB in different cell lines to explore the possibility of its use in anti-tumor therapy. The ability to flexibly control expression levels and timing should help us better understand the role of SpvB in pathogenesis and apoptosis induction. To this end, we have used a set of vectors and GFP-tagged *Salmonella* strains, recently developed in our laboratory, that drive the expression of heterologous proteins inside the eukaryotic cytoplasm when salicylate or acetyl salicylate is added to the cell culture [32–35]. The system combines a set of salicylate-regulated elements from *Pseudomonas putida* that work in a cascade, with a regulatory module integrated in the chromosome and an expression module in a plasmid. We demonstrate that upon salicylate induction, SpvB production depolymerizes F-actin in different tumor cell lines. Using a *purD* mutation, that prevents proliferation of the bacteria and protein expression inside the eukaryotic cell, we show that SpvB leads to a G1/S or G2/M arrest of the cell cycle and induces apoptosis via caspase-7 and -3.

## Materials and Methods

### Strain, plasmids and growth conditions

All plasmids and bacterial strains used in this work are described in Table S1. Cultures were grown aerobically at 180 r.p.m. and 37°C in Luria-Bertani (LB) medium and supplemented when necessary with ampicillin (100 μg/ml). Transductional crosses using phage P22 HT 105/1 *int201* [36] were used for transferring chromosomal markers among the strains. The transduction protocol has been described elsewhere [37].

### Cell cultures

HeLa (human cervix carcinoma cells), MCF-7 and MDA-MB-231 (human breast cancer cells), PANC-1 (human pancreatic adenocarcinoma cells) [38], HCT116 (human colorectal cancer cell) and RH-30 (human rhabdomyosarcoma cells) lines were a kind gift from J. Casadesús, A. López-Rivas D. Tuveson and J. Carvajal, respectively and previously obtained from the American Type Culture Collection (ATCC, Manassas, VA, USA). HeLa Kyoto cells expressing H2B-mCherry and mEGFP-α- tubulin [39] were provided by Dr D. Gerlich. Cells were cultured at 37°C in a 5% CO_2_ humidified incubator, and maintained in a Dulbecco's modified Eagle's medium (DMEM) (Sigma-Aldrich, Germany) supplemented with 2 mM L-glutamine and 10% heat-inactivated fetal bovine serum (FBS) containing a Penicillin-Streptomycin mixture (PAA laboratories GmbH, Austria).

### Molecular biology general procedures

All DNA manipulations were performed following standard protocols [40]. The *spvB* gene was amplified by PCR using total genomic DNA from *Salmonella* 14028 strain and primers spvBF1 (5'-cgtatcatatgttgatactaaatgg-3') and spvBR1 (5'- atatgatatcctatgagttgagtac-3') that contain the NdeI and EcoRV restriction sites, respectively. The 1.78-kb NdeI- EcoRV fragment was cloned in pMPO52 [34] into the same restriction sites. The resulting plasmid pMPO1036 contained *spvB* gene preceded by T7 SD sequence under the control of Pm promoter. 

Additionally, we cloned the 1.844 kb SmaI and SalI fragment from pMPO1036 into the same restriction sites of pMPO60 [34], generating the low-copy number version pMO1044. This plasmid possesses the *nasF* attenuator to reduce its basal expression level.

To generate SpvB C-terminally fused to the HA epitope, *spvB* gene was amplified by PCR using total genomic DNA from *Salmonella* 14028 strain with primers spvBF1 and spvBSalIR (5'- gatgtcgacgctgagttgagtaccctc-3') that contain the NdeI and SalI restriction sites, respectively. The 1.775-kb NdeI-SalI fragment was cloned in pMPO1004 into the same restriction sites, generating the plasmid pMPO1612.

### Construction of deletion mutants in *Salmonella* strains

For the construction of *spvB* and *purD* mutant strains, the "One Step Deletion" approach was used to replace target genes by the kanamycin resistance cassette [41]. The primers used for the amplification of kanamycin resistance gene from pKD4 were antispvBfin-P1 (5'- gggatccacaatttacaatgctttcgattttgagtttagtatttggagggtgtaggctggagctgcttc-3') and SpvBprinP2 (5'-ctttcctgccaaaagggggcaaggcgctgagtcagtcaggccctgacggccatatgaatatcctccttag-3'), or antifinpurD-P1 (5'-ccagcgcggtggcgcacagtacgcggccgccgctggtcaacacgcggtcgtgtaggctggagctgcttc-3') and purDprin-P2 (5'-ccgcgttgcagaacgtggctatcggcgtcaccgatattccggcgctgctgcatatgaatatcctccttag-3') for *spvB* and *purD* gene deletion, respectively. After the generation of primary mutants, the Δ*spvB* mutation was transduced by P22 phage into a fresh 14028 strain bearing the regulatory module of the salicylate inducible cascade expression system in its chromosome (MPO95). The *kan* gene was subsequently deleted using pCP20 and then, Δ*purD::kan* mutation was transduced by P22 phage into the Δ*spvB* deletion strain (MPO302) to generate the double mutant strain MPO325. 

### In vitro bacterial infection of tumor cells

Infection experiments were performed essentially as described elsewhere [34,42] with minimal modifications. Cells were cultured in 24-well plates at a density of 10^5^ cells per well 20 hours before infection. An overnight *Salmonella* culture was diluted 1:33 on fresh LB supplemented when necessary with ampicillin (100 μg/ml) and incubated at 37°C during 3.5 hours. Cells were equilibrated for 30 min in Earle’s buffered salt solution (EBSS) (Sigma-Aldrich, Germany) before infection. Bacteria were added at multiplicity of infection (m.o.i.) of 100:1-250:1 allowing the infection to proceed for 15 min at 37°C and 5% CO_2_. Wells were washed twice with Phosphate Buffer Saline (PBS) and incubated for 1 hour with DMEM containing 100 μg/ml gentamicin (PAA laboratories GmbH, Austria) to kill extracellular bacteria. When using Δ*purD* mutants, the medium also contained 366 μM adenine hemisulfate salt (normal adenine concentration). After that, the antibiotic concentration was reduced to 16 μg/ml, and 2 mM sodium salicylate (Sigma-Aldrich, Germany) was added to the culture medium to induce the SpvB expression. Adenine concentration was reduced 40-fold 1 hour after induction, to avoid bacterial proliferation (low adenine concentration). This adenine concentration was selected because was the highest concentration tested that did not allow the bacterial proliferation. Cells were incubated in this medium (DMEM containing salicylate and, when necessary, low adenine) until analysis. 

In order to reach higher infection rates, a m.o.i. of 250:1 was used in cell death assays and Western blot analysis.

### Invasion and intracellular replication assays

Different tumor cell lines (10^5^ cells/well) were infected with MPO302 or MPO325 *Salmonella* strains at m.o.i. 100:1, as described before. 3 hours after salicylate induction, cells were washed with PBS and treated with trypsin. After centrifugation, the cell pellet was resuspended in 400 μl of PBS and analyzed by a FACSCalibur flow cytometer (Becton Dickinson, Franklin Lakes, NJ. USA). 10,000 events were analyzed using CellQuest software (Becton Dickinson) for each sample. 

### Fluorescence microscopy

For fluorescence staining of F-actin, infected cells were grown on glass coverslips (12 mm, Thermo Scientific) and cell samples were taken 2-20 hours after induction and rinsed twice with PBS. Cells were subsequently fixed in 3.7% paraformaldehyde for 10 min at room temperature, and permeabilized in 0.1% Triton X-100 for 10 min. Thereafter, cells were briefly washed twice with PBS and incubated for 15 min with PBS containing Hoechst 33258 (1μg/ml) and 1:100 phalloidin-rhodamine (R415; Invitrogen Molecular Probes, USA) at room temperature in the dark. After washing with PBS (three times), the coverslips were mounted on slides and visualized with a confocal microscope Leica SPE (630X) or a fluorescence microscope Leica DMI4000B (320X) (Leica Microsystems GmbH, Wetzlar, Germany). 

### Cell death assays

Cells were seeded onto a 24-well plate (Nunc, Denmark) at a density of 5x10^4^ cells per well and were infected with *Salmonella* strains (14028, MPO302, MPO305 and MPO325) at a multiplicity of infection of 100:1 as described before. 24 hours after salicylate induction, culture supernatants were collected for analysis (experimental release sample). Cell death was determined by measuring lactate dehydrogenase (LDH) release in confluent infected cultures using CytoTox 96 non-radioactive cytotoxicity assay kit (Promega, USA), according to the manufacturer. The percentage of cytotoxicity was calculated as 100 x [(experimental LDH release − spontaneous LDH release)/ (total LDH release − spontaneous LDH release)], in which spontaneous LDH release was the level detected in the supernatant of an uninfected non-confluent HeLa cell culture. Total release is the activity in infected HeLa cell lysates. 1 μM staurosporine (STS) (Sigma-Aldrich, USA) was used as a control of cell death. 

### Cell cycle analysis

Cell distribution was determined by flow cytometry of propidium iodide (PI)-stained nuclei at the indicated times points after induction. The harvested cells (~5x10^5^ cells) were washed twice with PBS and fixed in 80% cold ethanol at -20°C for at least 24 hours. After fixation, cells were washed twice with PBS containing 0.1% BSA and the pellets were resuspended in phosphate-citrate buffer (0.2 M Na2HPO4, 0.1M citric acid pH 7.8) for 5 minutes at RT. After centrifugation at 500 *g* for 5 min, the cell pellet was resuspended in DNA staining solution (100 μg/ml of RNase A (R5125; Sigma-Aldrich), 40 μg/ml of PI, 0.1 mM EDTA pH 8 and 0.1% Triton X-100, in PBS) and incubated 30 min at 37°C in the dark. Samples were analyzed by flow cytometry. 10,000 events for each sample were analyzed using CellQuest software to determine the relative DNA content based on the presence of a red fluorescence and to evaluate the percentages of cells in sub-G1 (apoptotic cells), G0/G1, S, and G2/M phases. Results were presented as mean ± SD. Statistical analysis was performed using Student's *t* test. p < 0.05 was regarded as statistically significant.

### Live-infected cells imaging

HeLa Kyoto cells stably expressing H2B-mCherry and mEGFP-α-tubulin were plated on 35-mm glass bottom culture dishes (P35GC-1.5-10-C; MatTeK) and infected at m.o.i 100:1 with *Salmonella* MPO325 bearing pMPO61 or pMPO1044, as described before. After 1 hour of incubation in DMEM containing 16 μg/ml gentamicin, normal adenine concentration and sodium salicylate, the culture medium was replaced by 3 ml of Leibovitz’s L-15 medium (Sigma-Aldrich) supplemented with 2 mM L-glutamine and 10% FBS, and containing 16 μg/ml gentamicin, a low adenine concentration, and 2 mM sodium salicylate. Live-cell imaging was performed under Deltavision widefield microscope systems (Applied Precision, Issaquah, WA) equipped with a thermostat (37°C). Images were acquired using a 60x objective every 30 minutes for 20 hours as an image stack of 16- X 1-μm z-planes with 2 X 2 binning and analyzed using ImageJ software.

### Analysis of the ability of *Salmonella* deletion mutants to produce and translocate proteins into live eukaryotic cells

HeLa cells were infected with *purD* ^+^ (14028 and MPO302) or *purD* ^-^ (MPO305 and MPO325) strains bearing pMPO1004 as described previously. 24 hours post-infection, cells were detached by trypsin treatment, washed twice with PBS and then resuspended on 100 μl lysis buffer [43] and kept on ice for 30 minutes. The cell lysate was centrifuged at 20,000 g for 10 minutes and the supernatant was transferred to new tubes. Protein concentrations in the supernatant were determined by the RC DC Protein Assay protocol (BioRad, USA) and 30 μg of protein from the supernatant were loaded for 12.5% SDS-PAGE. After electrophoresis and Western blotting, immunoreactive products to anti-HA (1:5000 dilution) were detected by the SuperSignal West Dura Extended Duration Substrate reagents, according to the manufacturer's protocol (Pierce Protein Research Products, Thermo Scientific, USA).

HA antibody was purchased from Covance (USA) and α-tubulin antibody from Calbiochem (USA).

### Caspase and PARP Western blot analysis

HeLa and MCF-7 cells previously infected with MPO325 bearing pMPO52 or pMPO1036 were induced as described before. 24 hours (HeLa) or 48 hours (MCF-7) after induction, cells were collected by trypsin treatment, combined with cell culture media containing floating cells, and centrifuged. The cell pellet was washed with PBS and lysed in Urea lysis buffer (7M Urea, 2M Thiourea, 4% CHAPS, 40 mM DTT, protease inhibitors) on ice for 30 minutes. The cell lysate was centrifuged at 20,000 g for 10 minutes. Supernatant protein concentration was determined by the RC DC Protein Assay kit (BioRad, USA). Equal amounts of protein (50-100 μg/lane) were subjected to SDS-polyacrylamide gel electrophoresis under standard conditions. After electrophoresis and Western blotting, immunoreactive products to anti-caspase-3 (1:500 dilution), anti-caspase-7 (1:1000), anti-PARP (1:1000), anti-DnaK (1:5000) and anti-α-tubulin (1:10,000) were detected as above. Anti-α-tubulin antibody was used to ensure equal protein loading per lane. Antibodies against PARP (#9542), caspase-3 (#9662, #9664) and caspase-7 (#9492) were obtained from Cell Signaling Technology (USA). Anti-Dnak (mAb, 8E2/2) was obtained from Enzo Life Sciences (USA). Anti-mouse (A9044)- and anti-rabbit (A8275)- secondary antibodies were obtained from Sigma-Aldrich (Germany).

## Results

### Controlled expression of SpvB by *Salmonella* in eukaryotic cells depolymerizes F-actin and induces cell cycle arrest

We have utilized different salicylate inducible expression vectors to produce the SpvB protein by *Salmonella* in the cytosol of HeLa and MCF-7 cells and to analyze the effects of its controlled production. The producing *Salmonella* strain MPO302 is a *spvB* deletion mutant which bears in its chromosome the regulatory module of the salicylate inducible cascade expression system and a P*tac*-*gfp* cassette, to track the bacteria [34]. Briefly, in the regulatory module, constitutively expressed NahR is activated by salicylate binding and promotes *xylS2* transcription from Psal. In turn, XylS2 enhances the transcription of heterologous genes from the Pm promoter in the expression module. In addition, this system includes a terminator/antiterminator (*nasF*/*nasR*) circuit, from *Klebsiella oxytoca*, designed to reduce basal expression levels to detection limits [32,44].

**Figure 1:**
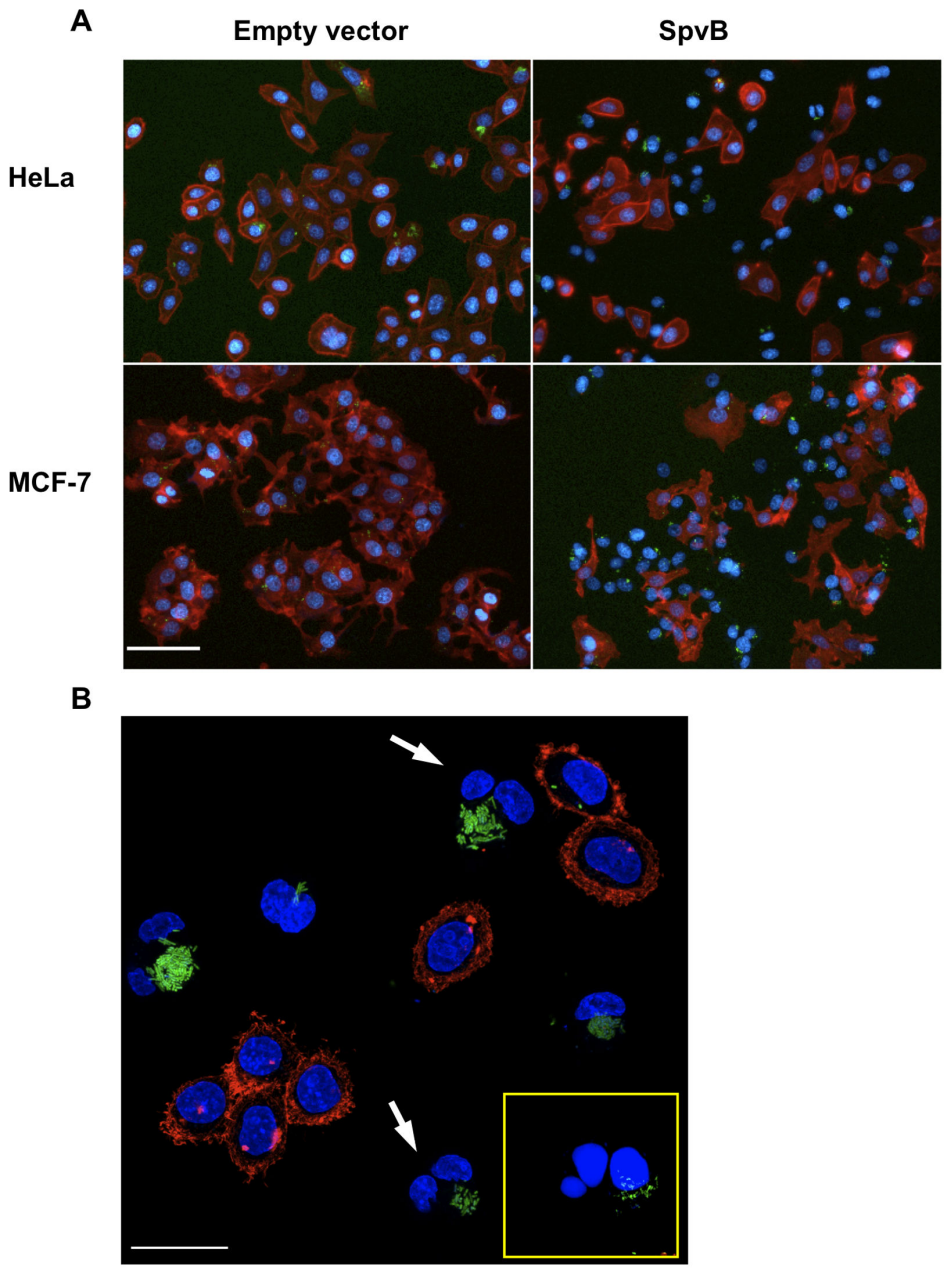
Effect of salicylate-dependent SpvB intracellular overexpression in tumor **cells**. (A) Fluorescence microscopy analysis of HeLa or MCF-7 cells infected with *Salmonella* MPO302 (Δ*spvB*) bearing the high-copy plasmids pMPO52 (empty vector) or pMPO1036 (SpvB) 4 h post-induction (320x). Scale bar: 50 μm. The MPO302 strain constitutively expresses GFP (green). The cells were simultaneously stained with phalloidin-rhodamine for polymerized actin (red) and Hoechst 33258 to stain eukaryotic and bacterial DNA (blue). (B) Higher magnification of infected HeLa cells culture 20 h post-induction using the Confocal Microscope Leica SPE (630x). Many infected cells show two nuclei (arrows) and some present fragmented nuclei (yellow box inset). Scale bar: 20 μm.

We first constructed the plasmid pMPO1036, which contains the *Salmonella spvB* coding sequence cloned downstream of the Pm promoter and the strong T7 ribosome binding site (RBS) sequence, that achieves high translation levels [34]. The *Salmonella* strain MPO302, carrying either the plasmid or the empty vector pMPO52, was used to infect HeLa and MCF-7 cells. Once the invasion was established, SpvB expression was induced with salicylate and cell cultures were analyzed by fluorescence microscopy 4 hours after induction. As shown in Figure 1A and Table 1, most HeLa and MCF-7 cells infected with SpvB-expressing *Salmonella* have lost their actin filaments 4 hours after salicylate induction (85% and 73% respectively), as determined by phalloidin-rhodamine staining and fluorescence microscopy. When cultures were analyzed 20 hours after induction, these cells showed a drastic decrease in proliferation (data not shown) and the frequency of infected cells without actin filaments increased to 99% in the case of HeLa, while MCF-7 showed little change (Table 1). Consistent with the observed depolymerization of F-actin cytoskeleton, Hoechst staining revealed a failure in cytokinesis, as 50% of infected cells contained two nuclei in both cell lines 20 hours after induction (arrows in Figure 1B and Table 1). Hoechst staining also detected the presence of cells with fragmented nuclei, suggesting that filament depolymerization and cytokinesis failure could lead to apoptosis induction. 

**Table 1:** Percentage of infected and binucleated cells lacking actin.

**Cell line**	**% of infected cells**	**% of infected cells without actin**	**% of binucleated infected cells without actin**
HeLa 4 h	61.33 ± 6.20	85.41 ± 7.11	9.17 ± 3.39
HeLa 20 h	55.38 ± 16.60	98.99 ± 2.11	48.01 ± 13.16
MCF-7 4 h	90.86 ± 3.23	72.75 ± 9.98	3.33 ± 1.14
MCF-7 20 h	78.46 ± 7.39	75.96 ± 9.69	57.94 ± 9.34

Data were obtained by counting ~ 100 cells per field 4 and 20 h post-induction. Values are means ± standard deviations of five independent fields.

Next, we tested the ability of SpvB to depolymerize actin when expressed from derivatives of pWSK29 [45], a stable low copy vector used in animal experiments. To that end, we cloned *spvB* in salicylate-regulated pWSK29 derivative vectors bearing the T7 RBS, as well as the *nasF* attenuator between the promoter and the *spvB* gene to reduce basal transcription [34]. We observed a similar effect on actin depolymerization and accumulation of binucleated cells 4 and 20 hours after salicylate induction, which indicated that sufficient SpvB production can be achieved even with a low copy number vector. Figure S1 shows the results 4 hours after salicylate induction using low copy number plasmids. 

### Salmonella *purD* mutant do not induce cell death per se but can be induced to produce protein inside the eukaryotic cell

To further explore the consequences of the controlled expression of SpvB in eukaryotic cell cultures by *Salmonella*, we analyzed its effect on cell death. Apoptosis is a relatively late epithelial cell response to *Salmonella* infection [12,13] and to characterize it, the infected cells must be maintained in cultures for at least 48 hours. Since *Salmonella* proliferation itself and the production of certain proteins during the first 7-8 hours of infection cause host cell death [12,13,46,47], in order to evaluate the SpvB overexpression effects, intracellular bacterial growth and effector proteins production must be restricted. It has been shown that a *purD* mutant is invasion proficient but unable to proliferate intracellularly once inside the eukaryotic cell and this proliferation deficiency can be temporally suppressed by adding adenine to the culture medium [48]. Therefore, we generated a *purD* mutation in the previously described *Salmonella* strain MPO302, to get the strain MPO325. In this way, intracellular proliferation and SpvB production would be controlled by the amount of adenine and the presence of salicylate in the culture medium respectively. 

In order to assess the suitability of this mutant, we first compared its infection capacity with that of its isogenic *purD* ^*+*^ strain infecting HeLa and MCF-7 cells. Quantification by flow cytometry analyses shown in Table 2 indicates that both strains have similar infection efficiencies. 

**Table 2:** Invasion and intracellular replication efficiency of *Salmonella* strains in tumor cells.

**Cell line**	**MPO302 Δ*spvB***	**MPO325 Δ*spvB*Δ*purD***
HeLa	65,06 ± 3,32	61,73 ± 1,35
MCF-7	77,90 ± 1,64	72,72 ± 2,32

Infected cells with a multiplicity of infection of 100:1 were harvested 4 h post-infection and 10,000 events were analysed by flow cytometry. Values represent percentages of infected cells (green fluorescent cells) and are the means ± standard deviations of three independent experiments.

**Figure 2:**
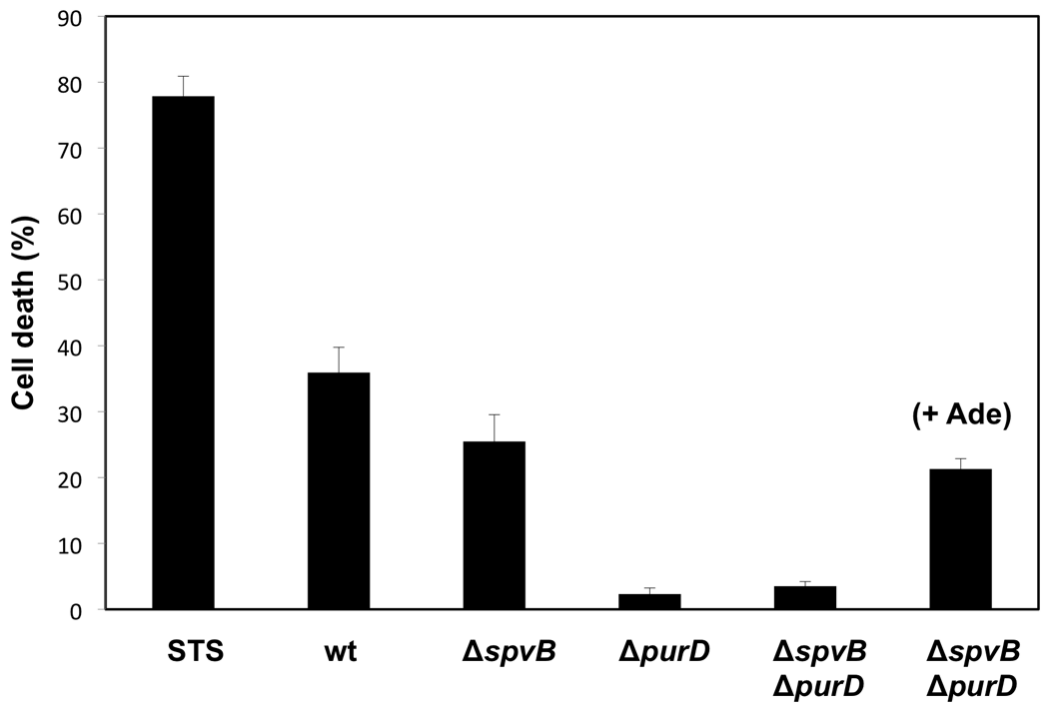
Cytotoxicity of *purD* mutant on HeLa **cells 24 h post-infection**. The cell death induction capacity of *Salmonella* strains was measured by the LDH release method. Staurosporine (STS) was used as a positive control for cell death. All samples were compared with the level of spontaneous cell death in non-infected cells. The results are the mean ± SD of three independent experiments. Culture medium was supplemented for two hours with normal adenine concentration and then was replaced by a low adenine concentration medium, except the sample on the right, which was supplemented with normal adenine concentration (+Ade). The multiplicity of infection was 100:1.

Secondly, we determined the intrinsic capacity of the *purD* mutant to induce cell death when infecting cell cultures in the presence of normal or low concentrations of adenine, by measuring cytosolic lactate dehydrogenase (LDH) release (Figure 2). We performed LDH assays with the *purD* ^*+*^ and *purD* ^-^ strains in different genetic backgrounds and two adenine concentrations, as described in materials and methods. *purD* ^*+*^ and *purD* ^-^ strains were used in HeLa infection experiments in which the culture medium was supplemented with the normal concentration of adenine either for 24 hours or for 2 hours, followed by 22 hours with a low adenine concentration. This adenine concentration was selected because it was the highest concentration tested that did not allow for bacterial proliferation in a *purD* ^-^ background (data not shown). As a control, the LDH release of a culture infected with a *purD* ^-^ strain with a normal adenine concentration was also tested. The results clearly demonstrated that although the wild type and *spvB* mutant strains are able to induce cell death when cultured with low concentration of adenine, the *purD* mutant is unable to produce cell death under the same conditions. However, when adenine was present in the medium at higher concentration, the cell death induction of the *purD* ^-^ was similar to that of the isogenic *purD* ^*+*^ strain. This behavior of the *purD* mutant was independent of the presence or absence of the Δ*spvB* mutation. These results confirm that the adenine requirement of the *purD* mutant prevents its induction of cell death. LDH release experiments using MCF-7 cells showed similar results (data not shown). 

**Figure 3:**
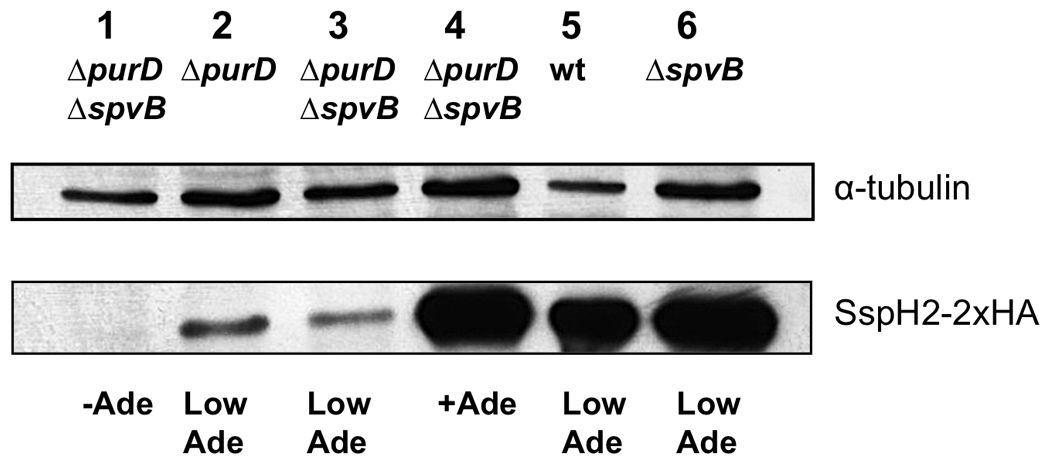
Protein induction and secretion of wt, *∆purD* and/or *∆spvB* strains. HeLa cells were infected with *Salmonella* strains in the presence of normal concentration of adenine. 2 h after salicylate induction, the adenine concentration was limited in lanes 2, 3, 5 and 6 (Ade) and normal in lane 4 (+Ade). Sample in lane 1 served as a control with no adenine added (-Ade). Strains in lane 1: MPO325; 2: MPO305; 3: MPO325; 4: MPO325; 5: 14028; 6: MPO302.

Finally, we examined the capacity of this mutant to produce and translocate proteins into the eukaryotic cytosol in response to salicylate under the conditions that restrict bacterial growth. To that end, we used an expression vector that carries the signal peptide of the virulence factor SspH2 fused to the HA epitope downstream of the Pm promoter. This vector allows the expression and translocation of the SspH2-HA fusion protein by *Salmonella* TTSS-2 in the presence of salicylate [34]. *purD* ^*+*^ and *purD* ^-^ strains bearing this plasmid were used in HeLa infection experiments in which the culture medium was supplemented with the two concentrations of adenine described above. As expected, the *purD* mutants required the normal concentration of adenine in the medium to obtain a production and secretion of protein equivalent to strains *purD* ^*+*^ and were unable to produce or secrete when adenine was absent (Figure 3). However, the condition that limited the adenine concentration 2 hours after infection still allowed detection of the salicylate-induced production and secretion of proteins. Finally, this experiment also showed that the presence of Δ*spvB* allele does not affect the SspH2-HA production or translocation. 

Taken together, these results indicate that the *purD* mutation and the use of appropriate adenine concentrations allow the induction of gene expression in response to salicylate while restricting bacterial proliferation and intrinsic induction of cell death caused by bacterial infection. Therefore, the conditional expression system in the *purD* ^-^ background used here represents a useful tool to study the effect of SpvB or other virulence factors in *Salmonella* infected cells independently of those produced by the normal bacterial proliferation during the infection cycle. 

### Effect of the regulated intracellular SpvB expression in a *purD* mutant

**Figure 4:**
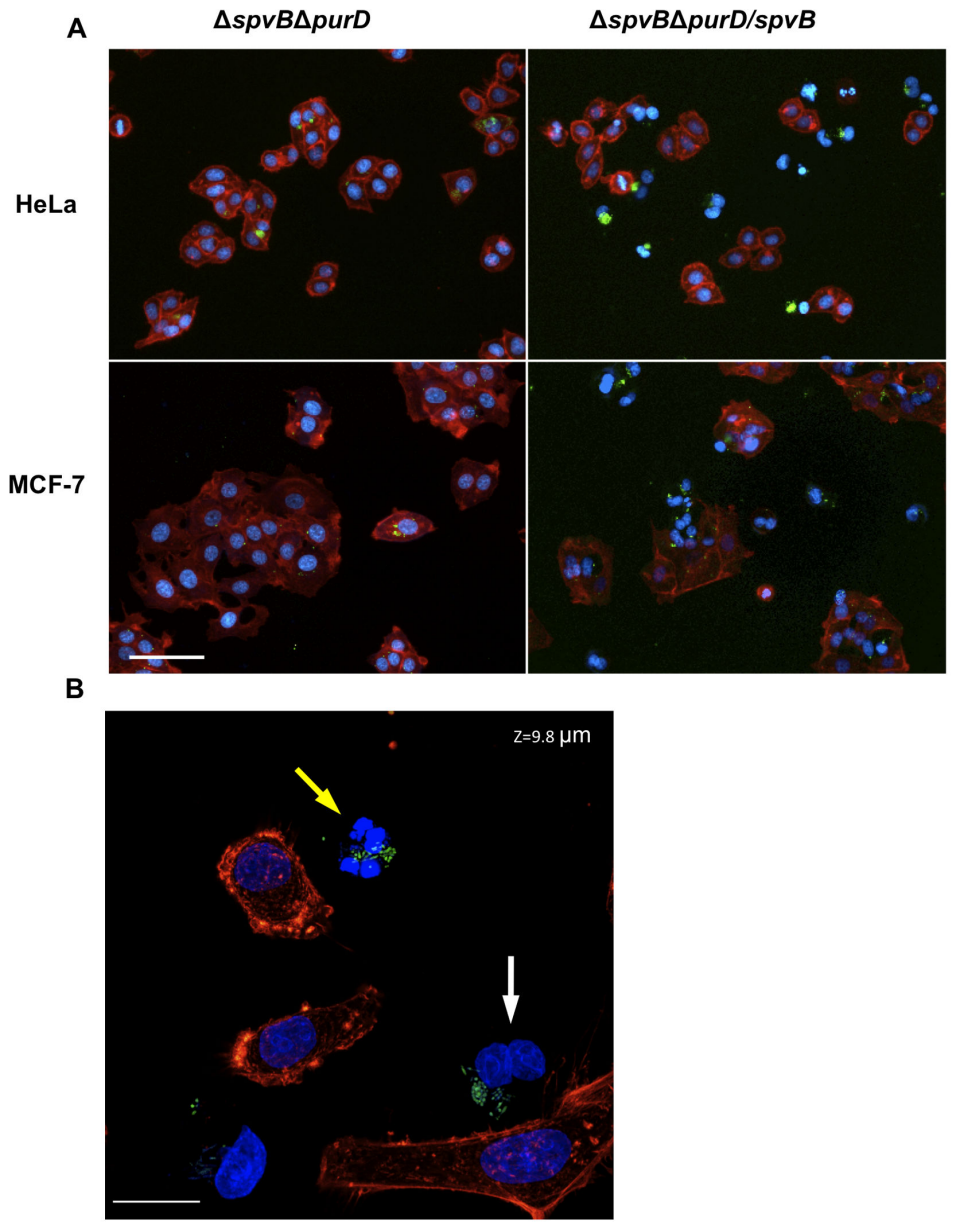
Cytoskeleton dismantling by intracellular overexpression of SpvB in *Salmonella* MPO325 (Δ*spvB*Δ*purD*). A) Merge images of HeLa and MCF-7 cells infected with *Salmonella* MPO325 bearing the empty vector (pMPO52) or the high-copy RBST7-SpvB plasmid (pMPO1036) and induced with salicylate for 20 h (320x). *Salmonella* constitutively express GFP (green). Eukaryotic nuclei and bacterial DNA were Hoechst stained (blue) and polymerized actin was phalloidin-rhodamine stained (red). Scale bar: 50 μm. B) Details of HeLa cells infected with MPO325/pMPO1036 20 h post-induction using the Confocal Microscope Leica SPE (630x). The infected cells lacking actin show double nuclei (white arrow) or fragmented nuclei (yellow arrow). Scale bar: 20 μm.

Having established the appropriate genetic background and culture conditions for protein production, we tested the ability of this strain to reproduce the effects of the SpvB overexpression under non-proliferating conditions. To this end, we infected HeLa and MCF-7 cells with *purD* ^-^ *Salmonella* strains carrying either the high copy vector overproducing SpvB (pMPO1036) or the empty vector (pMPO52). The culture medium was supplemented with normal concentration of adenine for 2 hours to allow the infection establishment and protein expression. Subsequently, the adenine concentration was reduced to avoid bacterial proliferation and the expression of bacterial proteins involved in cell death induction. The cell cultures were analyzed by fluorescence microscopy 4 and 20 hours after salicylate induction. The results (Figure 4) indicate that the *purD* ^-^ strain was able to reduce cell proliferation, induce actin depolymerization and the accumulation of binucleated cells after SpvB overproduction as proficiently as the *purD* ^*+*^ strain (compared to Figure 1) despite the absence of bacterial proliferation. As shown previously, we observed binucleated cells and fragmented nuclei. 

**Figure 5:**
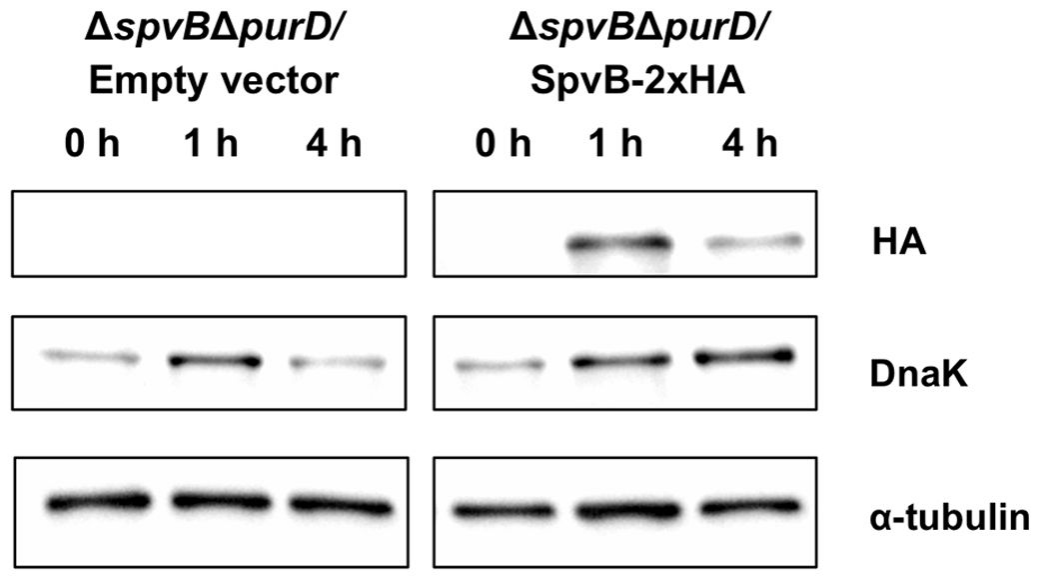
Induced expression of SpvB-HA in tumor cells. HeLa cells were infected with *Salmonella* MPO325 bearing the empty vector (pMPO52) or the plasmid producing SpvB-2xHA (pMPO1612) and induced by salicylate for indicated times under the conditions that restrict bacterial growth as described previously. Whole cell extracts were subjected to Western blotting to detect HA epitope fused to SpvB (68 kDa). The bacterial chaperone DnaK (70 kDa) was used as a control to confirm the presence of bacteria inside the tumor cell. Equal loading of the samples was confirmed by α-tubulin (60 kDa).

To prove the induced expression of SpvB within the eukaryotic cell, we performed a time course for the amount of this protein in infected HeLa cells. We first constructed the plasmid pMPO1612, which has the same features as the pMPO1036 vector but, in this case, SpvB is tagged with two copies of the HA epitope. We infected HeLa cells with *purD* ^-^ *Salmonella* strains as above, carrying either the empty vector or the plasmid pMPO1612 and determined the presence of the SpvB-2xHA protein by Western blot analysis using an anti-HA antibody. As shown in Figure 5, the presence of the tagged protein can be detected 1 and 4 hours after induction. Finally, we tested that the expression of this fusion protein also induces depolymerization of F-actin filaments and double nuclei accumulation by fluorescence microscopy (data not shown).

Therefore, these conditions make it possible to assess the specific role of the SpvB effector in cell death and apoptosis induction independently from bacterial infection.

### SpvB regulated production triggers cell cycle arrest in G1/S, G2/M and cell death

**Figure 6:**
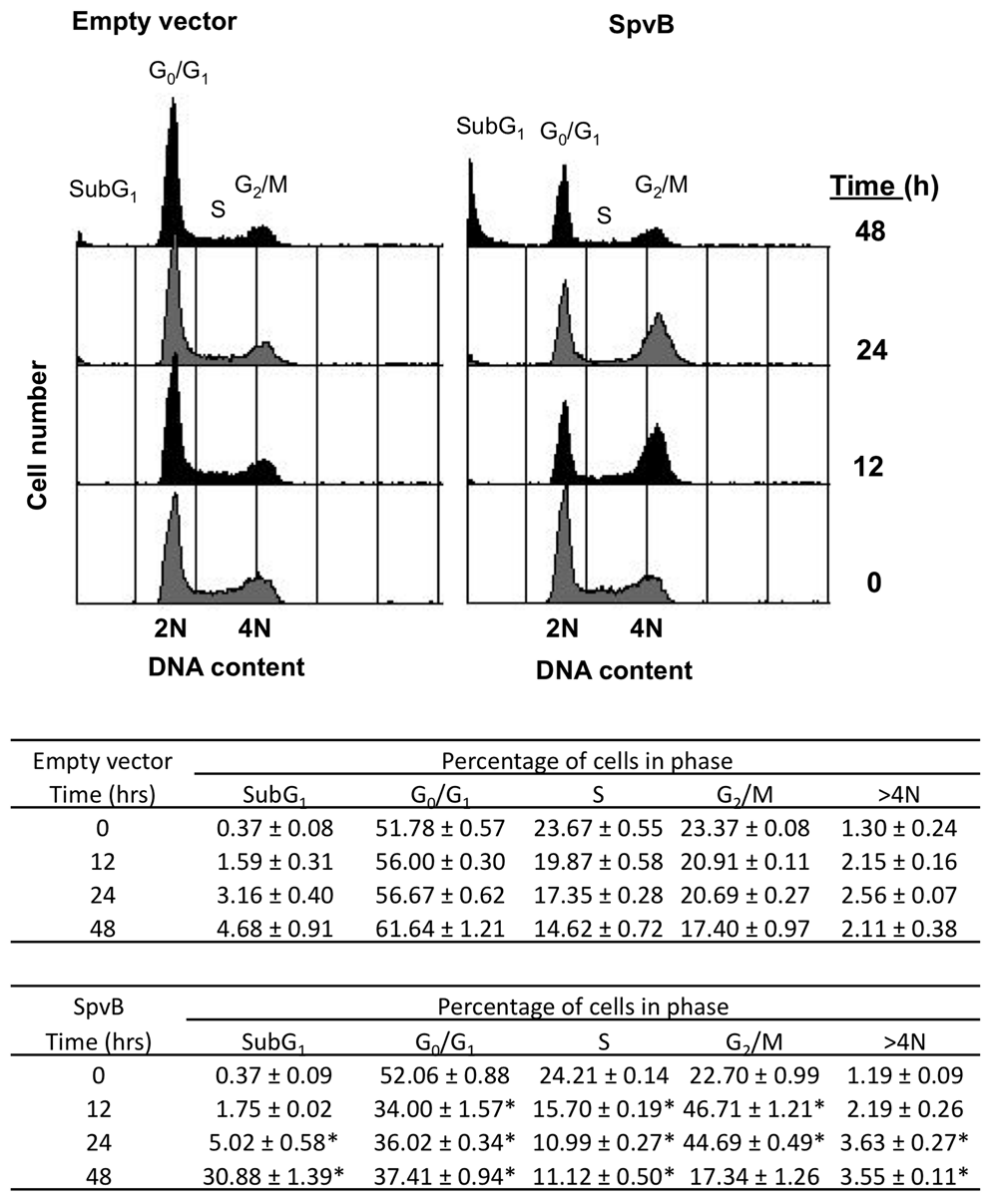
SpvB overproduction leads to cell cycle arrest in G1/S, G2/M and cell death. Cell cycle distribution of HeLa cells infected with MPO325 bearing pMPO52 (empty vector) or pMPO1036 plasmid (SpvB) at multiplicity of infection 250:1. The cells were harvested at the indicated times post-induction; 10,000 events were analyzed by flow cytometry for each sample. DNA content is represented on the x-axis; the number of cells counted is represented on the y-axis. Graphics are representative of three independent experiments. Data represents mean ± SD of three independent experiments. (*p < 0.05, Student's *t* test).

To further characterize the effect on cell cycle progression and cell viability upon SpvB controlled expression, we infected HeLa cells using the *purD* ^-^ mutant following the conditions described above. Cells were harvested 0, 12, 24 and 48 hours after induction of SpvB expression, and cell cycle progression was monitored by flow cytometry analysis (Figure 6). SpvB induced expression inside eukaryotic cells resulted in an increase of cells arrested with 4N DNA content, which was accompanied by a decrease in the number of cells with 2N DNA content. The 4N arrested cells reached almost 50% of the total population 12 hours after induction. Interestingly, we detected a necrotic/apoptotic sub-G1 population that increased (30% at 48 hours) while the arrested population decreased, showing that actin depolymerization and cell cycle arrest could lead to cell death. The percentages of cells in G0/G1 or S phases of the cell cycle decreases 12 hours after induction and then maintains or increases slightly.

**Figure 7:**
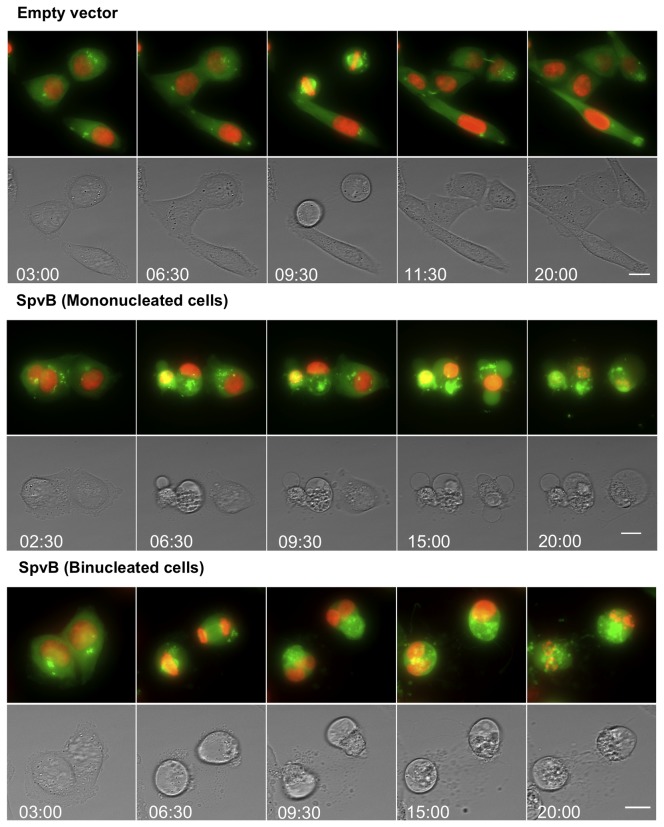
Confocal time-lapse imaging of infected cell with *Salmonella* MPO325 overproducing SpvB. HeLa cells expressing H2B-mCherry (red) and mEGFP–α-tubulin (green) were infected with *Salmonella* MPO325 bearing the empty vector or the plasmid producing SpvB and induced as in previous figure. Each cell behavior was monitored along the infection. Time is in hours. Maximum intensity projection of 16 *z* dimension slices. Scale bar: 15 μm.

Flow cytometry indicated that the 4N cell population decreases from 45% to 17% in the last 12 hours, whilst the subG1 peak increases. This decrease was not observed in the 2N cells (although we cannot discard the possibility that some of these cells are also dying due to the absence of F-actin), therefore, the most likely interpretation is that the 4N arrested cells eventually induce cell death. These 4N cells may correspond to binucleated cells, observed previously by microscopy analysis, which had already completed mitosis but that actin depolymerization made them unable to undergo cytokinesis (G1/S arrest), and mononucleated cells that after replicating their DNA, cannot enter mitosis and separate their chromatids for the same reason (G2/M arrest). To determine whether only one population of cells or both lead to cell death, we infected HeLa cells expressing both histone H2B-mCherry and mEGFP-α-tubulin with the *purD* mutants as above and analyzed the cell cycle progression by time-lapse microscopy. The results show that both mono- and binucleated arrested cells induce cell death when infected with *Salmonella* overexpressing SpvB (Figure 7 and Movies S1, S2 and S3). Therefore, our results demonstrate that SpvB depolymerizes actin, consequently preventing both G1/S and G2/M transition and that blocking either of these transitions inhibits cell proliferation and induces cell death.

### SpvB regulated expression induces activation of caspases-3 and -7

**Figure 8:**
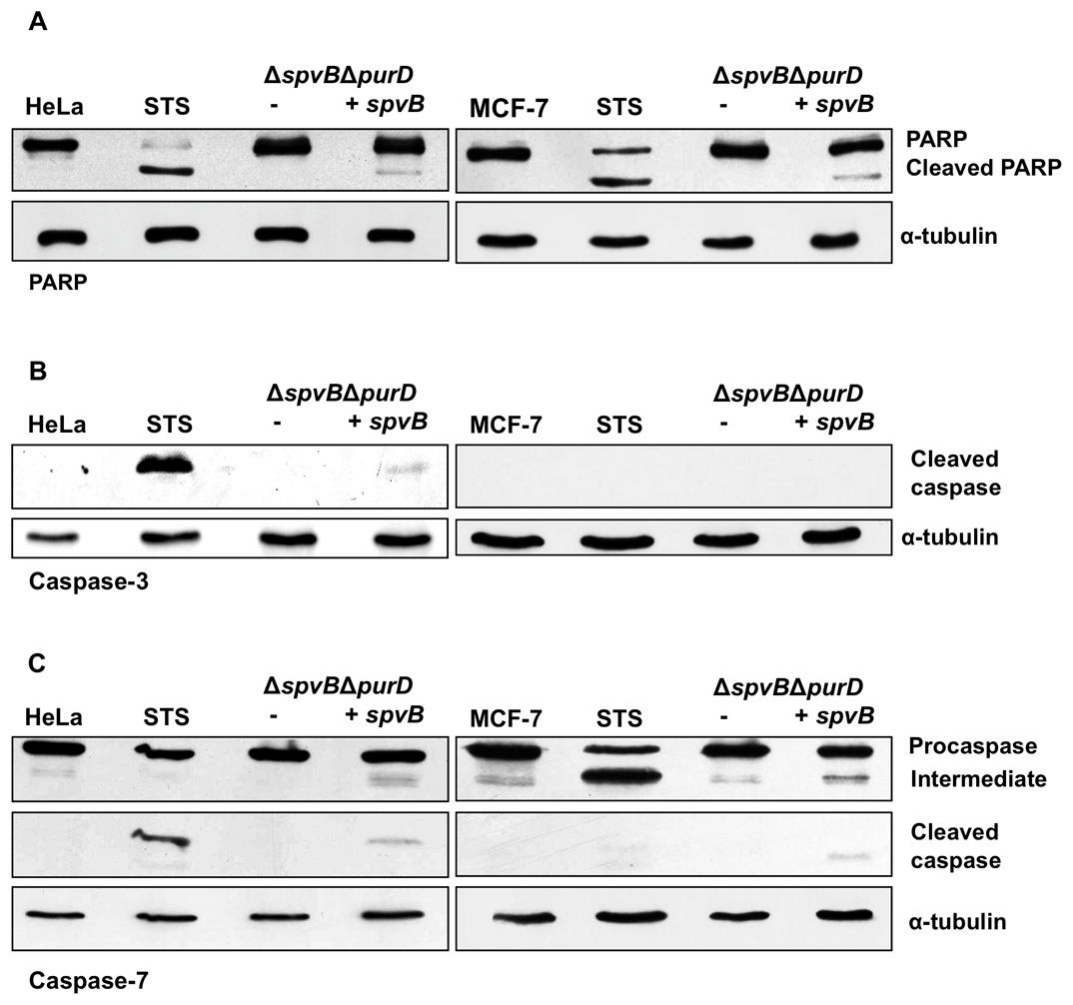
Apoptosis induction by overproduction of SpvB.

SpvB is one of the proteins required by *Salmonella* to induce caspase-3 dependent apoptosis in eukaryotic cells [13,16]. We wondered whether regulated expression of SpvB in conditions in which *Salmonella* is neither proliferating nor expressing other proteins could result in apoptotic cell death as suggested by the nuclei fragmentation and membrane blebbing produced. To that end, HeLa and MCF-7 cells were infected as previously described and harvested 24 or 48 hours after salicylate induction. The induction of apoptosis was analyzed by detecting the cleavage of PARP, a caspase substrate, by immunobloting (Figure 8A), following staurosporine treatment or expression of SpvB. This result was additionally confirmed by analyzing the activation of the effector caspase-3. As shown in Figure 8B, cleaved active caspase-3 was detected following SpvB expression in HeLa cells using an antibody specific for the cleaved caspase. MCF-7 cells lack caspase-3 [49], as confirmed by absence of cleaved caspase-3 following staurosporine treatment (Figure 8B). Therefore, we searched for the presence of active caspase-7 in the same samples. Figure 8C shows that SpvB expression increased the active form of this caspase in HeLa and MCF-7 cells. These results show that induction of SpvB overexpression can induce apoptosis in both tumor cell lines even where *Salmonella* proliferation is restricted and does not require capase-3, at least in MCF-7 cells. 

HeLa and MCF-7 cells were infected with *Salmonella* MPO325 bearing the empty vector or the plasmid producing SpvB and induced for 24 or 48 h, respectively. Whole cell extracts were prepared and assessed by Western blotting for A) PARP: full-length PARP (116 kDa); cleavage product (89 kDa); B) cleaved caspase-3 (17/19 kDa); C) caspase-7: pro-caspase-7 (35 kDa); intermediate pro-caspase-7 (32 kDa); cleaved caspase-7 (11 and 20 kDa). Equal loading of the samples was confirmed by α-tubulin (60 KDa).

To investigate whether SpvB-induced cell death is dependent on caspase-3 and -7 activation, we pretreated HeLa cells with the caspase inhibitor z-VAD-fmk before SpvB expression induction. Surprisingly, we observed that SpvB-induced cell death, measured as sub-G1 population, was only slightly reduced by the caspase inhibitor although the PARP cleavage was reduced (Figure S2 A and B). Caspase inhibitors reduce by 27% the sub-G1 population of the cultures infected with SpvB-expressing *Salmonella* and 62% when cultures are treated with latruculin A. These findings resemble other systems in which caspase inhibitors are unable to prevent cell death [50–54].

### Effect of SpvB expression in different tumor cell lines

**Figure 9:**
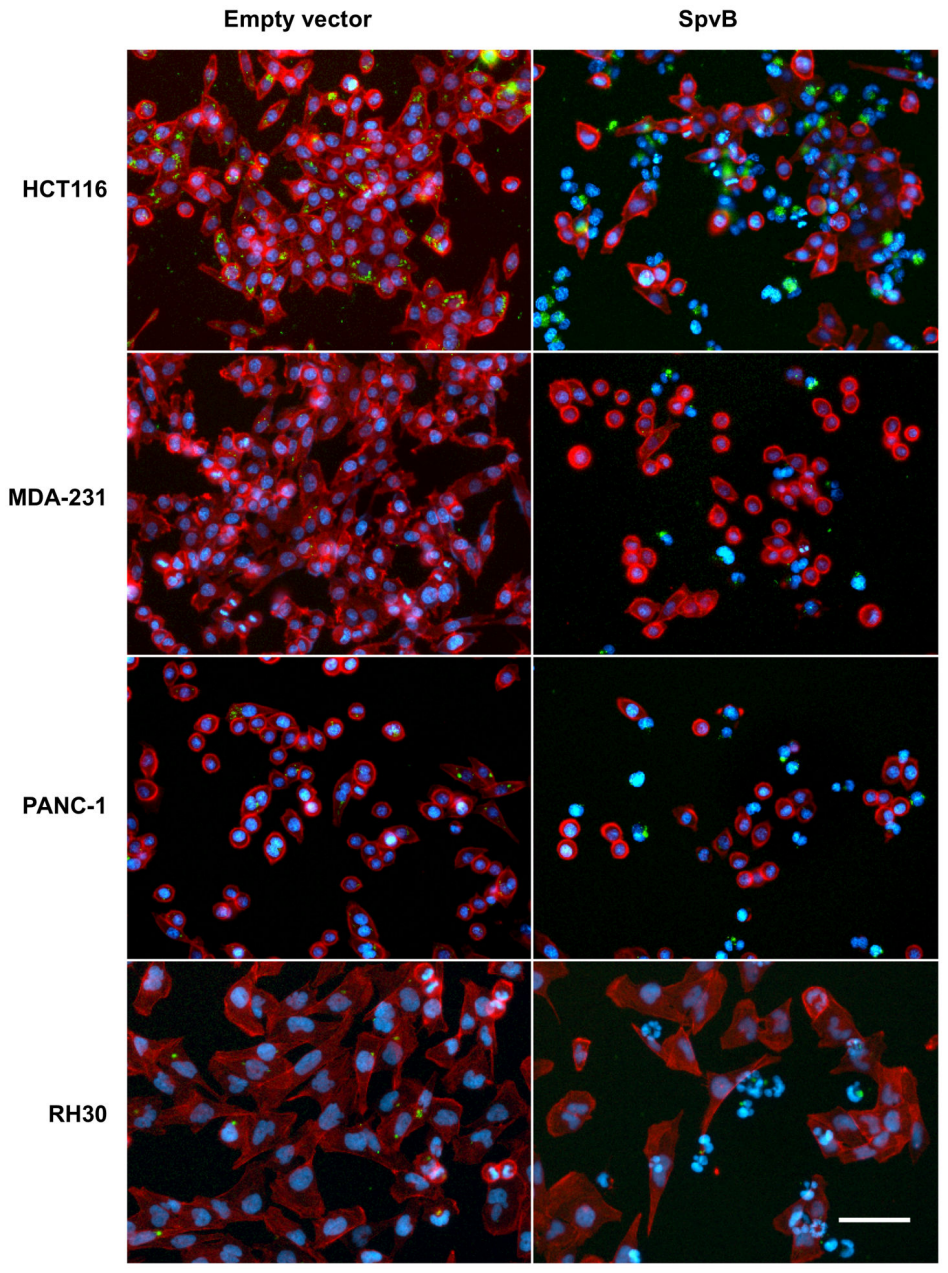
Salicylate dependent overproduction of SpvB in different tumor **cell lines**. HCT116, MDA-MB-231 and PANC-1 and RH30 cells were infected by *Salmonella* MPO325 bearing pMPO52 (empty vector) or pMPO1036 (SpvB) plasmid. The MPO302 strain constitutively expresses GFP (green). The cells were simultaneously stained with phalloidin-rhodamine for polymerized actin (red) and Hoechst 33258 to stain eukaryotic and bacterial DNA (blue). Scale bar: 50 μm. (320x).

To test whether expression of SpvB triggers the same response in different cell types, we analyzed the response of other three human tumor cell lines (MDA-MB-231, PANC-1 and HCT116 and RH30). These cells were infected as before with the *purD* mutant MPO325 bearing the vector that expresses SpvB upon salicylate induction or the empty vector as control. The consequences of SpvB expression were analyzed by fluorescence microscopy. As shown in Figure 9, 20 hours after induction, the F-actin cytoskeleton depolymerization was observed in all the cells lines tested and many cells undergo an SpvB-induced G1/S arrest, showing two closely associated HOECHST-stained nuclei. These results, as well as the presence of the apoptotic sub-G1 population, were confirmed by flow cytometry analysis (data not shown). In summary, SpvB induces F-actin depolymerization, cell cycle arrest and cell death in different tumor cell lines, suggesting that bacterially-delivered inducible SpvB expression might represent a powerful therapy against different types of tumors.

## Discussion

In this work we have used *Salmonella* for the ectopic expression of its virulence factor SpvB inside eukaryotic tumoral cell lines by using a salicylate inducible cascade expression system. These experiments have been carried out with three objectives: (i) to design a bacterial system of controlled delivery of virulence or other factors into the eukaryotic cytosol that allows characterization of their effects on the eukaryotic cell, (ii) to get insights into the role of SpvB in the infection process, and (iii) to exploit the apoptosis induction capacity of SpvB for potential applications in cancer therapy. 

Once *Salmonella* has infected a eukaryotic cell, bacterial proliferation leads to apoptosis induction and cell death within 18-24 hours due to the expression of certain effector proteins during the first 7-8 hours of infection. Therefore, to test the role of specific virulence factors or the effects of a cytotoxic protein it is necessary to prevent the production of such effectors. An attenuated *purD* mutant strain of *Salmonella* requires adenine for growth and cannot proliferate or express proteins inside the eukaryotic cells. However, adding a limited concentration of adenine can control proliferation of this strain within the cells. Adenine limitation can reduce or prevent bacterial proliferation but this can in turn reduce or prevent heterologous protein production. In fact, heterologous expression was not evident when adenine was absent from the culture medium (Figure 3). However, we have found that when infecting in the presence of a normal concentration of adenine for two hours followed by incubation of the cells in a medium with a low adenine concentration, this did not allow sufficient bacterial proliferation and protein expression to induce cell death but did allow significant heterologous protein production and secretion in the first hours (Figure 3). At least in the case of SpvB, production capacity of the system under these conditions was clearly higher than the normal SpvB production in the wild type bacteria (Figure 2), since salicylate-induction of SpvB expression triggered cell death even without bacterial proliferation or other proteins expression that were subsequently diminished by adenine limitation.

The regulatory system that combines the *purD* mutant and conditions that circumvent the deleterious effects of *Salmonella* proliferation have the potential of regulating the production and delivery of different factors into the eukaryotic cytosol and to analyze their effect on the cell physiology or viability. Therefore, they represent a powerful tool for analyzing the role of effector proteins in cell culture. This may also be of particular interest for analyzing the potential role of virulence factors from other pathogens in *Salmonella*.

Controlled expression of SpvB by *Salmonella* infecting either HeLa or MCF-7 cells led to a rapid loss of the actin cytoskeleton (Figure 1), an effect visible 4 hours after infection, followed by cell death. These effects are clearly associated with SpvB overproduction, since the infection with a wild type Salmonella strain does not lead to this rapid depolymerization of the F-actin filaments (data not shown) [16,18]. In addition, the *purD* mutant, which cannot proliferate inside the eukaryotic cells when adenine is limiting, cannot induce cell death when SpvB is produced from its native gene encoded in the chromosome (strain MPO305 in Figure 2) but can efficiently reproduce the phenotypes if SpvB production is induced by salicylate in the first hours after the infection, even in the absence of the endogenous copy of *spvB* (Figures 4, 6 and 7). Therefore, we can deduce that SpvB is responsible for cell death induction under these conditions although we cannot rule out that other *Salmonella* proteins produced in the first two hours are also contributing to the process.

Cell death features observed in this work suggest that, as previously reported, SpvB expression induces caspase-dependent apoptosis. These features include nuclei fragmentation, membrane blebbing, caspase activation and PARP cleavage induction after controlled SpvB expression. However, pretreatments with caspase inhibitors showed a slight decrease in cell death. In part, this could be due to an inefficient resolution of our system. In a cell culture, all the cells are sensitive to drug-induced apoptosis and caspase inhibitors whereas only a fraction of the culture are infected by *Salmonella* and affected by SpvB. With 50-60% of infected cells, we observed 20-30% of cell death in the whole cell culture 48 hours after SpvB expression induction. This percentage may be not high enough to detect a significant cell death reduction after treatments with caspase inhibitors. On the other hand, Kurita et al. (2003) have also shown that z-VAD-fmk inhibitors are unable to prevent DNA fragmentation induced in eukaryotic cells that express SpvB. The authors show that intracellular expression of SpvB in transfected Cos-7 cells caused DNA-ladder formation but addition of z-VAD-fmk inhibitors did not affect apoptosis induction in this system. Moreover, similar results have been previously obtained with other systems in which caspase are activated but the inhibitors are unable to reduce DNA fragmentation [50–54]. In some of these cases it have been suggested that alternative mechanisms of cell death, as autophagy, are induced in addition to apoptosis. Nevertheless, autophagy is an unlikely consequence of SpvB expression, since actin cytoskeleton is involved in this process [55,56]. 

The effect of SpvB on the cytoskeleton dynamics has been described previously, where transfection of mammalian CHO cells with vectors expressing *spvB* resulted in the loss of the cytoskeleton [22]. Moreover, the mechanism by which SpvB modifies G-actin monomers to prevent their polymerization in F-actin filaments has been well characterised *in vitro* (reviewed in 20). In addition, it has been reported that Spv proteins produced in the first few hours after infection are involved in apoptosis induction [13]. ADP-ribosylation of G-actin is an efficient strategy adopted by various toxins to disorganize the cytoskeleton of eukaryotic cells. A number of these drugs such as pectenotoxin have been shown to induce apoptosis and cell death since addition to cell cultures resulted in cell cycle arrest and apoptosis [28,30,31]. However, very little is known about the connection between this F-actin depolymerisation and cell death induction. 

The actin cytoskeleton, the cell cycle progression and apoptosis are interconnected processes, although the mechanisms governing this integration are not well understood. Many examples have shown that the state of the actin organization in the cell is critical for the cell cycle progression [29,57,58]. Therefore, the cell cycle blockage induced by SpvB might be the ultimate responsible for apoptosis induction and cell death. On the other hand, the actin structures formed in the cell are essential for other cellular processes besides cell cycle progression, such as the regulation of motility, cell shape, plasma membrane integrity or apoptosis itself [59]. Different drugs and regulatory proteins affecting actin dynamics have been used to show that actin can regulate the cell commitment to apoptosis in animal cells (reviewed in 60). It has been proposed that alteration of the actin dynamics itself, as the rate of polymerization/depolymerization, initiates or modulates the apoptosis signaling (for review see 61). Consequently, it seems likely that the actin depolymerization, and the subsequent cell cycle arrest, is also the condition that directly triggers the apoptosis due to SpvB production. 

Similar to other actin modifying toxins, production of SpvB resulted in cell cycle arrest 12 hours later [29,62]. However, we have shown that, while these toxins produce a G2/M cell cycle arrest, SpvB overexpression induces an arrest at both G1/S and G2/M transition phases, represented by the mono and binucleated 4N cell in Figures 6 and 7. Moreover, the SpvB-induced cell arrest may not be completely equivalent to that previously described since the percentage of endoreduplicated cells due to SpvB production, (represented by >4N in Figure 6) is clearly lower than that observed with other actin modifying toxins [29]. Binucleated cells could be arrested at G1/S transition phase of the cell cycle because the lack of actin prevents them from performing cytokinesis. In the same way, 4N mononucleated cells could be arrested after DNA replication due to the impossibility of activating mitosis in the absence of actin.

As cited above, it has been reported that SpvB is one of the proteins necessary for induction of apoptosis and that this is dependent on caspase-3. However, our results show that SpvB induction in MCF-7 cells, which lacks caspase-3, also induced programmed cell death in this cell line, which indicates that caspase-3 is not essential for this process. We have shown that the other major protease, caspase-7, is also activated during SpvB-dependent apoptosis induction in both HeLa and MCF-7 cells. The most plausible explanation is that caspase-7 takes control over the cell death program in the absence of caspase-3 and allows development of the process. Thus, although these caspases are functionally distinct proteases [63], they seem to be functionally redundant in the SpvB-induced apoptosis. Alternatively, caspase-7 may be the caspase actually involved in this process, regardless previous reports showing a connection to caspase-3.

The *Salmonella* infection and rapid apoptosis induction due to *Salmonella* SpvB overproduction is not restricted to HeLa or MCF-7 cell lines, but it is a general phenomenon observed in all tumor cell lines tested. Probably, actin dismantling is such a strong barrier to cell proliferation that no cell is able to overcome. 

Taken together, our results show that production of SpvB using the salicylate-inducible system in *Salmonella* in eukaryotic cells can be a potentially useful tool in anti-tumor therapy, regardless the tumor cell type. Thus, putative effector proteins with unknown function or any other cytotoxic protein could be analyzed following this procedure to determine its potential as cancer therapy agent.

## Supporting Information

Figure S1 **Effect of intracellular expression of SpvB from low-copy vectors in tumor cells.** Fluorescence microscopy analysis of HeLa or MCF-7 cells infected with *Salmonella* MPO302 (Δ*spvB*) bearing low-copy plasmids pMPO60 (control) and pMPO1044 (SpvB) after 4 h post-induction (320x). Scale bar: 50 μm. MPO302 strain constitutively expresse*s* GFP (green). The cells were simultaneously stained with phalloidin-rhodamine for polymerized actin (red) and Hoechst 33258 to stain eukaryotic and bacterial DNA (blue).
(TIF)

Figure S2 **Effect of the broad-spectrum caspase inhibitor z-VAD-fmk in cell death.** (A) Cell cycle distribution of HeLa cells treated with 4 μM latrunculin A (LatA, Sigma) or infected with *Salmonella* MPO325 strain producing SpvB for 48 h in the presence or absence of caspase inhibitor z-VAD-fmk (R&D Systems) at 40 µM concentration from 1 h prior to infection. Graphics are representative of three independent experiments. Data represents mean ± SD of three independent experiments. (B) Whole-cell lysates were subjected to Western blotting for full-length PARP (116 kDa) and cleavage product (89 kDa). Equal loading of the samples was confirmed by α-tubulin (60 kDa). LatA that disrupts cell cytoskeleton was used as a control.
(TIF)

Movie S1 **Time-lapse imaging of HeLa Kyoto cells infection with *Salmonella* bearing the empty vector.**
(AVI)

Movie S2 **Time-lapse imaging of HeLa Kyoto cells infection with *Salmonella* bearing the SpvB vector.** The movie shows arrested mononucleated cells death.
(AVI)

Movie S3 **Time-lapse imaging of HeLa Kyoto cells infection with *Salmonella* bearing the SpvB vector.** The movie shows arrested binucleated cells death.
(AVI)

Table S1 **Bacterial strains and plasmids used in this study.**
(DOCX)
