# Supplementary figures and images for: Novel Tools to Analyze the Function of Salmonella Effectors Show That SvpB Ectopic Expression Induces Cell Cycle Arrest in Tumor Cells
Source: PLoS One. 2013 Oct 21;8(10):e78458. doi: 10.1371/journal.pone.0078458 (PMC3804527; doi:10.1371/journal.pone.0078458)

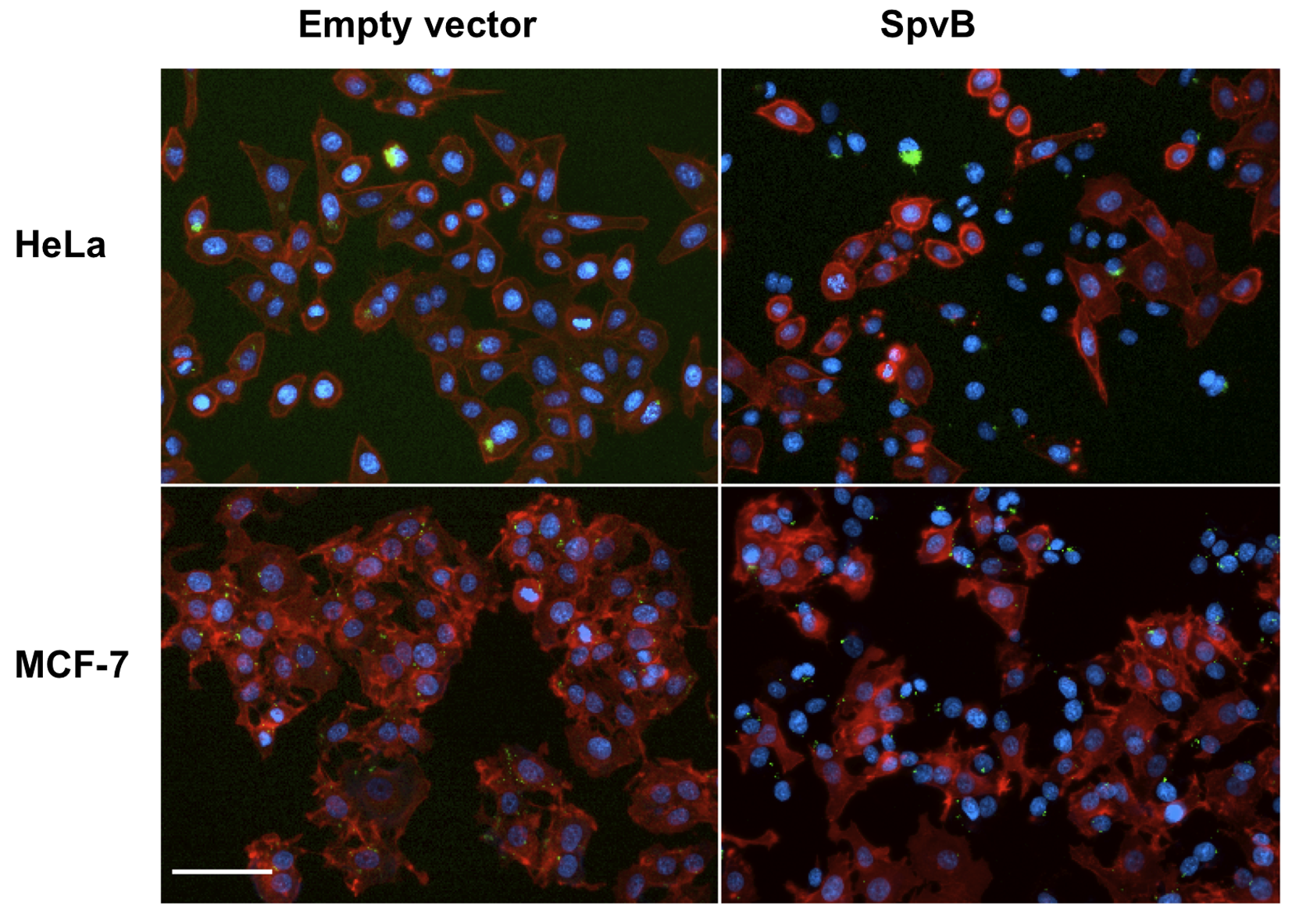

Supplement: Figure S1 — Effect of intracellular expression of SpvB from low-copy vectors in tumor cells. Fluorescence microscopy analysis of HeLa or MCF-7 cells infected with Salmonella MPO302 (ΔspvB) bearing low-copy plasmids pMPO60 (control) and pMPO1044 (SpvB) after 4 h post-induction (320x). Scale bar: 50 μm. MPO302 strain constitutively expresses GFP (green). The cells were simultaneously stained with phalloidin-rhodamine for polymerized actin (red) and Hoechst 33258 to stain eukaryotic and bacterial DNA (blue). (TIF) [file pone.0078458.s001.tif]

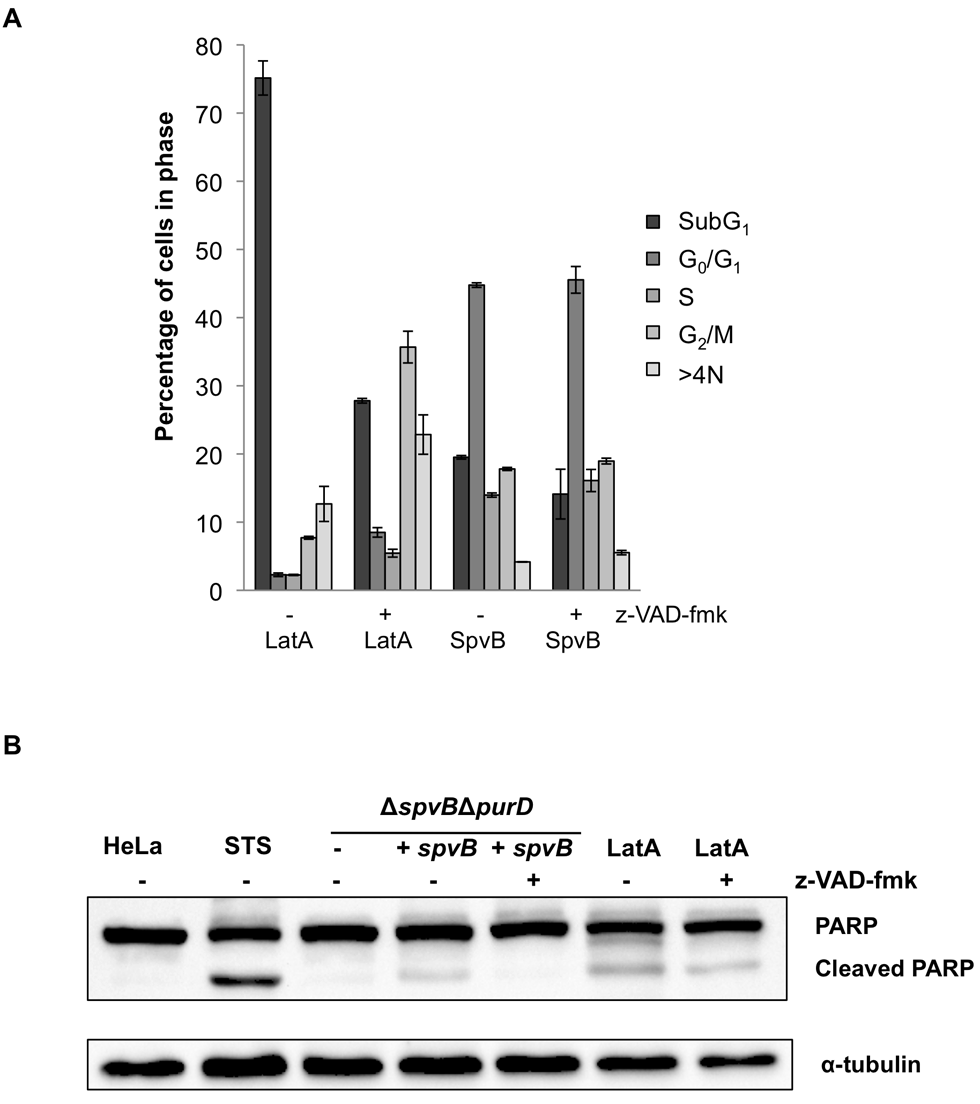

Supplement: Figure S2 — Effect of the broad-spectrum caspase inhibitor z-VAD-fmk in cell death. (A) Cell cycle distribution of HeLa cells treated with 4 μM latrunculin A (LatA, Sigma) or infected with Salmonella MPO325 strain producing SpvB for 48 h in the presence or absence of caspase inhibitor z-VAD-fmk (R&D Systems) at 40 µM concentration from 1 h prior to infection. Graphics are representative of three independent experiments. Data represents mean ± SD of three independent experiments. (B) Whole-cell lysates were subjected to Western blotting for full-length PARP (116 kDa) and cleavage product (89 kDa). Equal loading of the samples was confirmed by α-tubulin (60 kDa). LatA that disrupts cell cytoskeleton was used as a control. (TIF) [file pone.0078458.s002.tif]
