# Supplementary material for: Novel Tools to Analyze the Function of Salmonella Effectors Show That SvpB Ectopic Expression Induces Cell Cycle Arrest in Tumor Cells
Source: PLoS One. 2013 Oct 21;8(10):e78458. doi: 10.1371/journal.pone.0078458 (PMC3804527; doi:10.1371/journal.pone.0078458)
Supplement: Table S1 — Bacterial strains and plasmids used in this study. (DOCX) [file pone.0078458.s006.docx]

|  | **Characteristics** | **Reference** |
| --- | --- | --- |
| ***E. coli* strains** |  |  |
| *DH5α* | *deoR endA1 gyrA96 recA1 supE44* | [1] |
|  |  |  |
| ***S. typhimurium* strains** |  |  |
| 14028 | Wild type strain | [2] |
| MPO94 | 14028 ∆*trg:: nahR/*P*_sal_–xylS2-cat* /P*_Tac_–gfp* fusion | [3] |
| MPO95 | 14028 ∆*trg:: nahR/*P*_sal_–xylS2* /P*_Tac_–gfp* fusion | This work |
| MPO98 | MPO95 ∆*spvB::kan* | This work |
| MPO301 | MPO95 ∆*purD::kan* | This work |
| MPO302 | MPO98 ∆*spvB* | This work |
| MPO305 | MPO301 ∆*purD* | This work |
| MPO325 | MPO302 ∆*spvB*∆*purD::kan* | This work |
|  |  |  |
| **Plasmids** |  |  |
| pCP20 | Ap^R^, Cm^R^, Ts (30ºC) | [4] |
| pKD4 | Ap^R^, Km^R^, *oriRγ* | [5] |
| pKD46 | Ap^R^, *oriR101*, *repA101*(ts), *araBp-gam-bet-exo* | [5] |
| pMPO52 | Ap^R^, expression vector with rrnBT1T2-Pm-T7 SD sequence, ColE1 replication origin | [3] |
| pMPO60 | Ap^R^, expression vector with rrnBT1T2-Pm-*nasF* attenuator, M13 replication origin | [3] |
| pMPO61 | Ap^R^, expression vector with rrnBT1T2-Pm- *nasF* attenuator-T7 SD sequence-MCSII, MCS M13 replication origin | [3] |
| pMPO1004 | Ap^R^, expression vector with rrnBT1T2-Pm-T7 SD sequence- *sspH2* signal peptide-HA epitope encoding sequence, ColE1 replication origin | [3] |
| pMPO1036 | Ap^R^, expression vector with rrnBT1T2-Pm-T7 SD sequence*-spvB*, ColE1 replication origin | This work |
| pMPO1044 | Ap^R^, expression vector with rrnBT1T2-Pm-*nasF* attenuator- T7 SD sequence- *spvB*, M13 replication origin | This work |
| pMPO1612 | Ap^R^, expression vector with rrnBT1T2- Pm-T7 SD sequence-*spvB*- HA epitope encoding sequence, ColE1 replication origin | This work |

**References**

1. Hanahan D (1983) Studies on transformation of *Escherichia coli* with plasmids. J Mol Biol 166: 557-580.

2. Fields PI, Swanson RV, Haidaris CG, Heffron F (1986) Mutants of *Salmonella typhimurium* that cannot survive within the macrophage are avirulent. Proc Natl Acad Sci U S A 83: 5189-5193.

3. Medina C, Santero E, Gomez-Skarmeta JL, Royo JL (2012) Engineered *Salmonella* allows real-time heterologous gene expression monitoring within infected zebrafish embryos. J Biotechnol 157: 413-416.

4. Cherepanov PP, Wackernagel W (1995) Gene disruption in *Escherichia coli*: TcR and KmR cassettes with the option of Flp-catalyzed excision of the antibiotic-resistance determinant. Gene 158: 9-14.

5. Datsenko KA, Wanner BL (2000) One-step inactivation of chromosomal genes in *Escherichia coli* K-12 using PCR products. Proc Natl Acad Sci U S A 97: 6640-6645.
